# Volume Flow and Peak Systolic Velocity of the Arteriovenous Circuit in Patients after Percutaneous Deep Venous Arterialization

**DOI:** 10.3390/diagnostics10100760

**Published:** 2020-09-28

**Authors:** Michiel A. Schreve, Eline Huizing, Steven Kum, Jean-Paul P. M. de Vries, Gert J. de Borst, Çağdaş Ünlü

**Affiliations:** 1 Department of Surgery, Northwest Clinics, 1815 JD Alkmaar, The Netherlands; e.huizing@nwz.nl (E.H.); Cagdas.Unlu@nwz.nl (Ç.Ü.); 2 Vascular Service, Department of Surgery, Changi General Hospital, Singapore 529889, Singapore; stevenkum.dr@gmail.com; 3 Division of Vascular Surgery, Department of Surgery, University Medical Centre Groningen, 9713 GZ Groningen, The Netherlands; j.p.p.m.de.vries@umcg.nl; 4 Department of Vascular Surgery, University Medical Center Utrecht, 3584 CX Utrecht, The Netherlands; g.j.deborst-2@umcutrecht.nl

**Keywords:** chronic limb threatening ischemia, peripheral arterial disease, endovascular, venous arterialization, duplex ultrasound, peak systolic velocity, volume flow

## Abstract

Percutaneous deep venous arterialization (pDVA) is a developing technique for limb salvage in patients with chronic limb-threatening ischemia by creating an arteriovenous (AV) circuit. After pDVA, patency of the AV circuit is evaluated using duplex ultrasound (DUS) imaging. Peak systolic velocity (PSV) and volume flow (VF) values for maintaining a patent AV circuit are undefined; therefore, guidance about when a reintervention should be performed is lacking. The objective of this study was to interpret post-pDVA PSV and VF values in relation to AV circuit preservation. This was performed by analyzing DUS results of 22 post-pDVA patients. A total of 670 PSV and 623 VF measurements were collected. A PSV value of ≤55 cm/s and a VF value of ≤195 mL/min were found predictive for failure. The reliability of PSV and VF measurements in patent AV-circuits was good (intraclass correlation coefficient; PSV, 0.85; VF, 0.88). In conclusion, this study is the first to analyze DUS measurements in post-pDVA patients and showed that DUS can be used to anticipate for failure. The thresholds found can be used to help interpret DUS measurements in post-pDVA patients. More research in a larger patient population is needed to prospectively validate these thresholds.

## 1. Introduction

Chronic limb-threatening ischemia (CLTI) is the clinical end stage of peripheral artery disease (PAD), which is associated with severe ischemia and has an amputation risk of 25% at 1 year when untreated [1]. The cornerstone to prevent amputation in CLTI patients is the combination of medication and revascularization [2]. Endovascular interventions and bypass surgery are the most performed techniques for revascularization, but sometimes technically unsuitable due to extensiveness of the disease. As a result, up to 20% of patients with severe limb ischemia are unsuitable for bypass surgery or angioplasty [3].

For these patients, percutaneous deep venous arterialization (pDVA) could be an alternative technique for limb salvage. In this procedure, a connection is made between a tibial artery and a tibial vein creating an arteriovenous (AF) fistula, after which the valves in the vein are destroyed, and the side branches are covered with a covered stent to provide pressurized arterial flow to the venous system of the foot. We call this the arteriovenous (AV) circuit [4].

Duplex ultrasound (DUS) surveillance after the procedure is indicated to detect any inflow or outflow problems of the AV circuit. DUS measurements include peak systolic velocity (PSV), as recommended for regular infrainguinal bypasses, and volume flow (VF) measurements as done for AV fistulas [5,6]. However, determining when the AV circuit is at risk for occlusion or when a reintervention should be performed is difficult because DUS imaging criteria for failed and patent AV circuits are lacking. Therefore, the aim of this study was to interpret the post-pDVA PSV and VF values by specifying these values in a patent and failed AV circuit and by selecting optimal thresholds for detecting a stenosis or occlusion anywhere in the AV circuit.

## 2. Materials and Methods

This study was conducted according to the principles of the Declaration of Helsinki and approved by the Institutional Board of Directors of Northwest Clinics, Alkmaar, the Netherlands on 20 May 2016, and Changi General Hospital, Singapore in 2013, with reference code 2013/828/C. All patients provided written informed consent for the procedure.

### 2.1. Patient Selection

All consecutive patients treated by pDVA using the LimFlow device (LimFlow SA, Paris, France) between July 2014 and June 2018 for CLTI in the Northwest Clinics in Alkmaar and Changi General Hospital in Singapore were eligible for the present study. CLTI was defined as the presence of PAD in combination with gangrene, a lower limb ulceration >2 weeks’ duration, or rest pain with affirmative hemodynamic studies [2]. Patients without DUS measurements at follow-up due to early amputation or death were excluded.

Inclusion criteria for the pDVA procedure were Rutherford category ≥4, no angiographically evident distal target artery for endovascular therapy or a distal bypass, and at least 1 patent tibial artery in the proximal segment. Exclusion criteria were acute limb ischemia, extensive tissue loss or infection that precluded limb salvage, known deep vein thrombosis, allergy to aspirin or clopidogrel, and/or contraindication to anticoagulation [7]. Patient suitability for the pDVA procedure was assessed by an independent committee from Syntactx, including an experienced vascular surgeon and interventionalist.

### 2.2. pDVA Procedure

The procedure was performed using the LimFlow device, as described in detail previously [4,7]. In brief, antegrade arterial access and distal venous access were achieved by ultrasound-guided puncture of the femoral artery and target tibial vein at the ankle, respectively. The arterial and venous catheters were inserted and advanced to the crossing point. A needle from the arterial catheter was deployed to cross from the artery to the vein to create the AV fistula. A 0.14-inch guidewire was then passed through the needle into the vein all the way down to the foot. A valvulotome was inserted to lyse the valves in the vein.

Stent grafts were implanted in the vein from the level of the ankle toward the crossing point, which was in turn covered by a tapered self-expanding stent graft. The tapered crossing stent secures the AV fistula, and the stents in the vein and outflow in the foot are considered to be the AV circuit.

Postprocedure, patients were prescribed lifelong antiplatelet therapy (aspirin 100 mg or clopidogrel 75 mg) in combination with therapeutic low-molecular-weight heparin (LMWH) for at least 3 months. Both hospitals used the same procedure protocol and patient selection criteria.

### 2.3. DUS Measurements

All DUS measurements were performed in the hospitals by trained vascular ultrasound technologists. The measurements were done using Philips ultrasound scanners: Affiniti 70G in Amsterdam, The Netherlands, and IU22 at Changi General Hospital, Singapore.

The patient was examined supine with the hip of the measured leg rotated externally and the knee slightly flexed. A L12-3 linear array transducer was placed behind the knee in the transverse plane and moved distally along the posteromedial or anterolateral aspect of the calf to locate the popliteal artery, tibial vessels, stent, and anastomoses. Grayscale and color Doppler imaging was used to check the vessel lumen and flow direction in transverse and longitudinal views for any abnormalities. PSV and VF measurements were recorded with the vessels in longitudinal views using the duplex Doppler mode. A ≤60° Doppler angle with the cursor parallel to the vessel wall was used when the PSV was measured. The sample volume was positioned in the center and completely encompassed the vessel lumen. On the Doppler trace, the baseline was lowered and the velocity scale adjusted appropriately to avoid aliasing. A low wall filter setting was used. 

**Figure 1:**
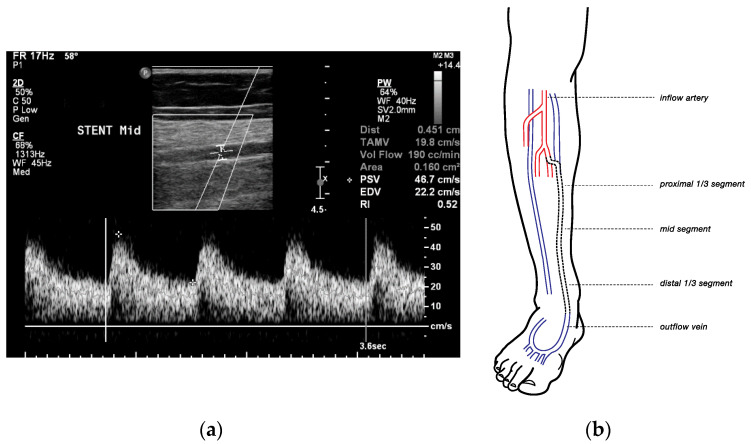
Image of duplex ultrasound measurement and measurement points. (**a**) Duplex ultrasound image of measuring the volume flow in the mid-segment of the stented vein. (**b**) Schematic image of the various duplex ultrasounds measuring points in patients after percutaneous deep venous arterialization.

To obtain the VF, the diameter of the vessel was measured with the calipers at right angles to the sample volume. Three pulse cycles on the spectral trace were selected, and the system automatically estimated the time-averaged mean and calculated the VF in milliliters/minute (Figure 1a).

Measurements consisting of diameter, PSV, and volume flow, were recorded at 5 points, over straight segments (Figure 1b):

- At the inflow artery (popliteal artery, P3)
- At the proximal one-third segment, middle segment, and distal one-third segment of the stented vein, and
- At the distal outflow vein, >3 cm distal to the lowest point of the covered stent (e.g., lateral plantar vein).

The surveillance protocol included measurements every 2 weeks for the first 2 months postprocedural and at 3-, 6-, and 12-months postprocedural, when possible. Deviations from the protocol and additional measurements were depending on the condition of the patient. Indications for additional measurements included aberrant findings on DUS, impaired wound healing, aberrant pain, or other signs of ischemia. Extra measurements were also performed after a reintervention.

### 2.4. Reinterventions and Digital Subtraction Angiography

The decision to perform a reintervention was left at the operator’s discretion and primarily based on the condition of the patient, e.g., pain, new or persistent wounds, or other signs of ischemia. When there was any doubt of the patency of the AV circuit on DUS, a digital subtraction angiography (DSA) was performed preemptively.

The angiograms performed during reinterventions were collected and stored for postintervention analysis in detail if the DSAs were performed ≤1 month after DUS. DSAs performed >1 month after DUS were not associated, because the time between the two imaging techniques was considered too long. Duplex values followed by a stenosis ≥50% seen on angiography or an occlusion as seen on DUS were marked as failed. If no stenosis ≥50% was seen on angiogram or if no occlusion was seen on DUS, DUS values were reported as patent.

### 2.5. Optimal Threshold Selection for Predicting ≥50% Stenosis or Occlusion

To determine a range of values that indicated a failed AV circuit (≥50% stenosis or occlusion anywhere in the AV circuit) and a patent AV circuit (absence of ≥50% stenosis or occlusion anywhere in the AV circuit), receiver operating characteristic (ROC) curves with corresponding sensitivity and specificity were calculated. Two cutoff values for each measuring point were selected. The first value with a specificity of >80% was selected as the cutoff point of the lowest value to ensure that false positives for occlusion were low. The first value with a sensitivity of >80% was selected for the cutoff point of the highest value to ensure that the false negatives for occlusion were low. In this way, it was possible to determine a low PSV/VF cutoff point, which indicated that under this specific value, flow problems are likely to occur, and to determine a high PSV/VF cutoff point, which indicates that flow problems are unlikely to occur.

### 2.6. Reliability of the PSV and VF Measurements

Test–retest analyses were performed to explore the reliability of the DUS measurements. Measurements were included in the analysis if they were considered patent, succeeded each other within 30 days, and if both PSV and VF measurements were performed during the same consultation. In this way, the chance for equal conditions between the two succeeded measurements and between PSV and VF measurements was considered highest. Consecutive measurements were performed using the same ultrasound scanners.

The analyses were performed using the intraclass correlation coefficient. Values <0.5 were considered as poor reliability, between 0.5 and 0.74 as moderate reliability, between 0.75 and 0.90 as good reliability, and values >0.90 were considered as excellent reliability [8].

### 2.7. Data Collection, End Points, and Definitions

Patient demographics, baseline risk factors, and PSV and VF measurements were retrospectively collected. Data were derived from electronic medical records, clinical records, and imaging reports. Follow-up visits for the patients were based on their clinical condition.

The primary outcome was the optimal thresholds for detecting stenosis of ≥50% or occlusion within the first 3 months postprocedure. Secondary outcomes were the reliability of the measurements, the mean of post-pDVA PSV and VF values in patent and failed AV circuits, and the predictive value of PSV and VF values for major amputation and wound healing.

The AV circuit consists of the inflow artery, the AV fistula, the stented vein, and the outflow veins in the foot. Major amputation was defined as amputation above the ankle [2]. Wounds were as assessed by the treating physician and considered healed if they were fully epithelized. Reintervention was defined as repeat percutaneous intervention. Reliability of the measurements was defined as the consistency of successive measurements.

### 2.8. Statistical Analysis

Statistical analysis was performed using SPSS software (version 23, IBM, Armonk, NY, USA). Quantile–quantile plots were analyzed to determine whether continuous variables followed a normal distribution. If the points in the quantile–quantile plot lie on a straight diagonal line, the data were defined as normally distributed. Normally distributed continuous variables are expressed as mean ± standard deviation. Nonnormally distributed data were presented as median with the interquartile range (IQR) and were log transformed to normally distributed data for comparison. Log-transformed normally distributed data were compared using the independent *t* test. The means calculated from log-transformed data were transformed back to normal values and are reported as geometric means. Categorical variables are expressed as numbers with percentages. 

Kaplan–Meier analyses were performed to estimate the amputation-free survival, defined as avoidance of major amputation (above the ankle) of the index limb or death (any cause) and wound healing at 12 months. Wounds were considered healed if they were fully epithelized. For patients who died before complete wound healing, the date of death was defined as the cutoff date. For patients who underwent major amputation, the time to wound healing was considered to be infinite [9].

Reliability analyses were performed using the intraclass correlation coefficient using the alpha two-way random effects model with absolute agreement. Single measures values were used. 

ROC curves were calculated to establish a threshold for PSV and VF values. Statistical significance was defined as *p* < 0.05.

## 3. Results

### 3.1. Patient Characteristics

Between July 2014 and June 2018, 27 patients underwent the pDVA procedure in the Changi General Hospital in Singapore (*n* = 19) and in the North West Clinics in Alkmaar, the Netherlands (*n* = 8). Of the 27 patients, 22 had DUS measurements at follow-up (from July 2014 to December 2019) and were included in the study. The other five patients were lost to follow-up because of an early amputation (*n* = 3), death (*n* = 1), or living abroad (*n* = 1). Patient characteristics are summarized in Table 1. Of all patients, seven patients were classified as Rutherford stage 6. These patients were eligible for the procedure as the ischemic ulcers or gangrene affected slightly more than just the digits of the foot and therefore did not deemed unsalvageable in the operator’s opinion. The target arteries included the posterior tibial artery (*n* = 14), tibioperoneal trunk (*n* = 4), anterior tibial artery (*n* = 2), and popliteal artery (*n* = 2). Target veins were posterior tibial vein (*n* = 18), tibioperoneal trunk (*n* = 1), anterior tibial vein (*n* = 2), and popliteal vein (*n* = 1).

Five patients (23%) required a major amputation, and six patients (27%) died during follow-up. Rutherford category 6, coronary artery disease, and renal insufficiency was found in three of the five patients requiring amputation. Reasons to perform a major amputation included a combination of new wounds and occluded graft (34 months postprocedural), an occluded graft and occluded femoral popliteal bypass (7 months postprocedural), infection ((*n* = 2), 6 and 9 months postprocedural), and worsening of tissue loss (1 month post procedural). The median time between the intervention and major amputations was 6.7 (3.4–21.3) months. The estimated amputation-free survival and wound healing at 12 months was 71.6% and 64.5%, respectively. The median follow-up was 5 months (IQR, 0.6–35 months). 

### 3.2. Reinterventions

A total of 47 DSAs were performed in 19 patients. Reasons to perform a DSA were pain, new or persistent wounds, preemptive, or stenosis or occlusion identified with DUS. Of these 47 DSAs, two AV circuits were found patent and one stenosis in the outflow vein was found and left untreated because of adequate foot perfusion and a healed wound. In the other 45 DSAs, the lesions found were located in the inflow arteries (*n* = 18), in the stented vein (*n* = 12), and, most often, in the outflow veins (*n* = 31). Of all lesions, 45 were stenoses (74%) and 16 occlusions (26%). Treatment modalities for thrombosis included thrombolysis with or without mechanical thrombectomy. Occlusions were treated with a thrombectomy device in combination with percutaneous transluminal angioplasty (PTA) using plain old balloon angioplasty (POBA) or drug eluting balloons (DEB). Stenoses were treated using POBA or DEB and stents when necessary. A stealing outflow vein was found twice and treated by embolization or ligation. 

The difference between the PSV and VF values before and after reintervention were statistically significant: the geometric mean PSV was 54 ± 2 cm/s before and 77 ± 2 cm/s after (*p* < 0.001), and the geometric mean VF was 121 ± 3 mL/min before and 178 ± 2 mL/min after (*p* = 0.005).

### 3.3. PSV and VF Measurements

The 22 patients had a total of 670 PSV and 623 VF measurements with a median of 27 PSV measurements (IQR, 8–91 measurements) and 25 VF measurements (IQR, 4–77 measurements) per person. Of these, 487 PSV and 464 VF measurements were reported as patent and 183 PSV and 159 VF measurements as failed. 

### 3.4. Test–Retest Reliability

The results of the reliability test of the PSV and VF values are shown in Table 2. Per measuring point, two consecutive measurements were used. The mean time between the measurements was 14 ± 8 days. The mean time point for first reliability measurement was at 36 days postprocedural. The ICC were found highest in the distal 1/3 segment and middle segment for the PSV and VF values, respectively.

### 3.5. Predictive Value for Failed AV Circuits

The diagnostic accuracy of the PSV values to predict failure anywhere in the AV circuit was highest for the measurements performed in the proximal one-third and middle part of the stented vein and for VF values at the inflow artery and middle segment in the stent (Table 3).

### 3.6. Optimal PSV and VF Threshold for Predicting Failed AV Circuits ≤3 Months Postprocedure

The optimal cutoff values for detecting a failed AV-circuit are summarized in Table 4. The accuracy was highest for the cutoff values in the proximal 1/3 segment and middle segment of the stented vein for the PSV values and in the inflow artery and middle segment of the stented vein for the VF values. 

## 4. Discussion

The pDVA procedure seems to be a promising option for patients with no-option CLTI [7,10]. Early detection of flow problems is required to prevent failure of the AV circuit. Current guidelines support DUS surveillance and prophylactic intervention for asymptomatic vein graft stenosis to promote long-term patency [2]. DUS PSV measurements have a sufficiently high sensitivity and specificity in the femoropopliteal region but poor correlation in the tibial vessels [11]. In venous access surgery, the dialysis AV shunt is monitored by DUS using PSV and VF measurements. A PSV value of 400 cm/s has good discrimination to predict >50% stenosis, and a threshold of a PSV value >500 cm/s will reliably identify graft-threatening lesions [12]. A VF value of <300 mL/min predicts failure of the graft [13]. The VF is measured in the artery, which creates a more reliable outcome because the diameter is predictable and the flow has less turbulence.

In this study, the accuracy of the cutoff points and the diagnostic accuracy was found highest for the measurements performed in the middle of the stented vein for both PSV and VF measurements. The variation between the measuring points was not expected to occur for the VF measurements, as the VF values are expected to be the same at every measuring point. The variation could be explained by the occurrence of turbulent flow within the AV circuit. VF is most accurately measured when laminar flow is present. Turbulent flow occurs when the direction of the flow is disrupted, which occurs by the curves in the crossing stent and in the veins in the foot. Flow in the middle and distal segments in the stent should therefore be the most laminar, and thus, the most accurate point to measure the VF, which was also found in the reliability tests. The measurements performed in the middle and distal segments in the stent had the highest intraclass correlation coefficient scores.

However, the reliability results found in the present study should be interpreted carefully as the values of the measurements were collected retrospectively and therefore not performed under strict protocol. In addition, some consecutive measurements could have been performed by different vascular ultrasound technologists, which preferably would be the same technician to correctly analyze the accuracy of the test–retest measurements. However, a recent study investigated the inter-rater reliability of VF measurements performed in the posterior tibial artery and found an ICC of 0.7 for mean VF values and an ICC of 0.87 for maximum VF values [14]. Thus, the effect of the various ultrasound technicians performing the measurements may be limited.

In this study, a low cutoff point was found to be predictive for failure. This is in line in bypass graft DUS surveillance studies that associated >70% stenosis with mid-graft PSV <45 cm/s [15,16] and PSV < 60 cm/s with flow problems [17]. In the present study, a cutoff point of ≤55 cm/s was found to be predictive for failure in the middle segment of the stented vein.

However, in most patients with vascular problems, high PSV values are used to indicate a stenosis and thus a problem. This is only possible when measured close to the stenosis. However, stenosis and occlusions are sometimes difficult to detect, and it would be more helpful to provide thresholds that indicates flow problems without the necessity to measure at a specific spot. This is important for patients after pDVA specifically, because flow problems that occur due to stenosis or occlusions occur more frequently in the inflow arteries or outflow veins of the foot and less frequent in the stented vein. The present study showed that the flow and the velocity of blood in the stented vein decreased because of a stenosis or occlusion in the inflow arteries or outflow veins in the foot. Therefore, the measurements in the stented vein are helpful to identify flow problems in the inflow arteries and outflow veins. The occurrence of a decrease in PSV values in patients with flow problems has also been reported in a recent study where the PSV values of patients with PAD and healthy controls were compared. At the anterior tibial artery, PSV values of 43.7 vs. 65.4 (*p* < 0.001) were found in patients with PAD and healthy controls, respectively, and comparable results were found when measured in the posterior tibial artery (43.4 vs. 74.1; *p* < 0.001) [18].

**Figure 2:**
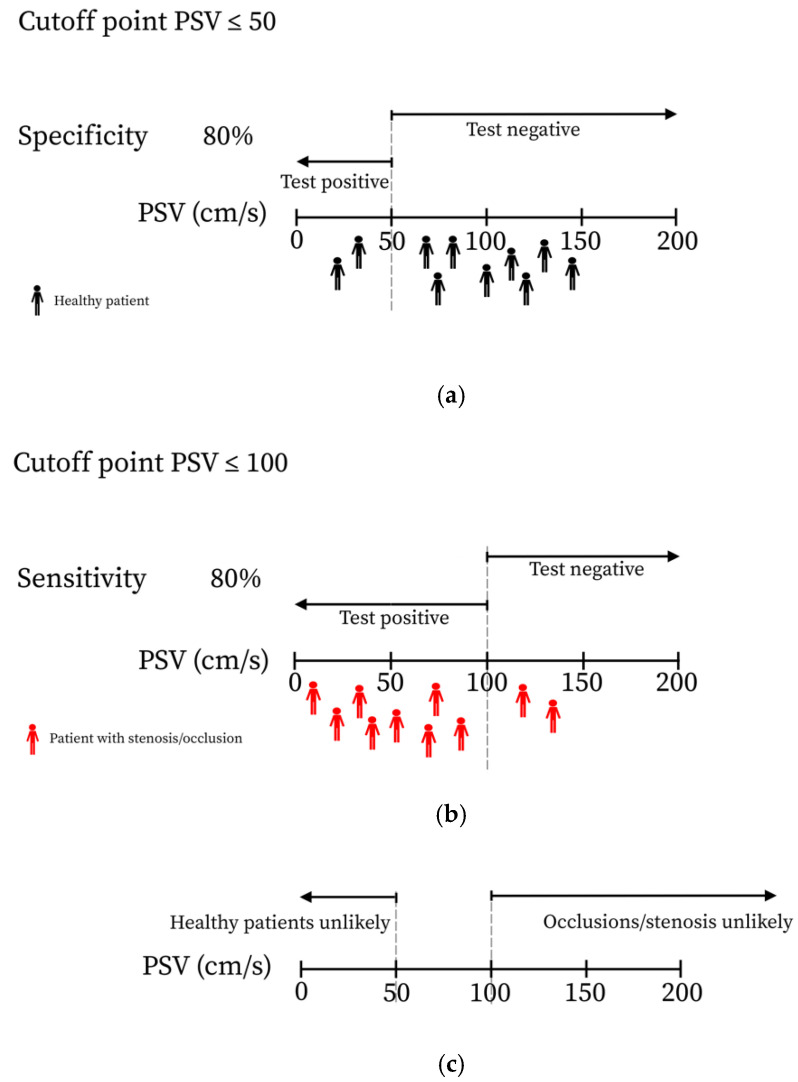
Illustration of the meaning of a high specificity and sensitivity for the selected cutoff points. (**a**) Illustrates that in 80% of all healthy patients, a peak systolic velocity (PSV) value of > 50 cm/s was found. (**b**) Illustrates that in 80% of the patients with an occlusion or stenosis, a PSV ≤ 100 cm/s was found. (**c**) Illustrates that taking into account figures (**a**) and (**b**), this means that if in a patient a PSV value ≤ 50 is found, it is unlikely that the patient is healthy and if in a patient a PSV value of >100 cm/s is found, the patient is unlikely to have an occlusion or stenosis.

The predictability of PSV and VF values for stenosis or occlusion was determined by ROC analysis. The lower cutoff point was selected according to a high specificity (>80%) to determine a point on which the PSV values below this point are unlikely to be found within healthy patients. The higher cutoff point was selected according to a high sensitivity (>80%) to determine a cutoff point in which PSV values higher than this point are unlikely to be found in patients with a stenosis or occlusion (Figure 2). In between these values, there is a gray area in which no hard conclusion can be made. The analysis showed that a VF value of <195 mL/min was predictive for failure and >364 mL/min was defined as patent measured in the middle of the stent. For a PSV value, <55 cm/s was found to be predictive for failure and >99 cm/s as an indication for patency measured in the middle of the stent. These values could be a helpful contribution for current clinical practice while waiting for more and prospective studies with a larger cohort of patients.

### Limitations

The present study is not without limitations. In this cohort, not every DUS value was compared with angiography. Therefore, DUS values could have been incorrectly considered as patent. In addition, as mentioned earlier, the interrater reliability was not assessed, leaving any bias in this area unknown. In addition, because of the novelty of the technique, the studied patient population is small, resulting in broad confidence intervals and less reliable results. Final, normal PSV, and VF of the bypass do not always represent a good perfusion of the foot. Side branches can evolve, which do not influence the flow in the AV circuit but give a poor distal foot perfusion.

## 5. Conclusions

This study is the first to analyze DUS measurements in post-pDVA patients. Because of the frequent occurrence of stenosis and occlusions in this specific patient population, there is a high need for more insight in DUS interpretation to detect failure of the AV-circuit and preserve the limb. This study showed that surveillance of the AV circuit can be performed by DUS to anticipate for failure, but the small sample size of the study does not allow firm conclusions to be drawn. One could consider the possibility of stenosis or occlusions that could occur when PSV values of <55 cm/s and VF values of <195 mL/min are found, but a final judgment about the perfusion of the foot and an indication for reintervention should be based on a combination between the clinical evaluation, the DUS findings with PSV and VF values, and transcutaneous oxygen measurements pending further research to prospectively validate these thresholds.

## Figures and Tables

**Table 1:** Baseline characteristics.

Variable	Values
Patients	22
Men	9 (40.9)
Age, years	67 ± 17
Comorbidities	
Hypertension	18 (81.8)
Diabetes	15 (68.2)
Hyperlipidemia	17 (77.3)
Cerebrovascular accident	4 (18.2)
Coronary artery disease	7 (31.8)
Dialysis dependent	2 (9.1)
Body mass index, kg/m^2^	22 ± 5
Laboratory results	
Creatinine, mg/dL	85 (66–145)
eGFR <30 mL/min/1.73 m^2^	5 (22.7)
Rutherford	
4	1 (4.5)
5	14 (63.6)
6	7 (31.8)
SVS WIfI risk staging	
Low risk	1 (4.5)
Moderate risk	5 (22.7)
High risk	16 (72.7)

Continuous data are presented as mean ± standard deviation or median (interquartile range), and categorical data are presented as number (%). eGFR, estimated glomerular filtration rate; SVS WIfI, Society for Vascular Surgery risk system based on Wound, Ischemia, and foot Infection.

**Table 2:** Reliability of consecutively measured peak systolic velocity (PSV) and volume flow (VF) values.

Measurement Point	PSV			VF		
	No.	ICC	95% CI	No.	ICC	95% CI
Inflow artery	8	0.747	0.17–0.94	5	0.367	−0.80 to 0.91
Proximal 1/3 segment	10	0.588	0.02–0.88	10	0.549	−0.80 to 0.87
Middle segment	10	0.652	0.13–0.90	10	0.875	0.58–0.97
Distal 1/3 segment	10	0.846	0.37–0.96	10	0.771	0.33–0.94
Outflow vein	10	0.354	−0.39 to 0.80	10	0.647	0.08–0.90

CI, confidence interval; ICC, intraclass correlation coefficient; No, number of patients included in the analysis.

**Table 3:** Area under the curve (AUC) values for evaluating failed arteriovenous circuits by the peak systolic velocity (PSV) and volume flow (VF) values of various measuring points.

Measurement Point	PSV				VF			
	No.	AUC	95% CI	*p*	No.	AUC	95% CI	*p*
Inflow artery	50	0.691	0.51–0.88	0.048	32	0.859	0.73–0.99	0.003
Proximal 1/3 segment	68	0.747	0.63–0.87	0.001	60	0.693	0.54–0.84	0.019
Middle segment	72	0.710	0.58–0.84	0.005	65	0.704	0.57–0.84	0.010
Distal 1/3 segment	69	0.707	0.57–0.84	0.006	66	0.644	0.49–0.80	0.068
Outflow vein	68	0.552	0.40–0.70	0.498	62	0.583	0.43–0.74	0.303

CI, confidence interval; No., number of measurements included in the analysis.

**Table 4:** Optimal cutoff point (COP) for evaluating failed arteriovenous circuits by the peak systolic velocity (PSV) and volume flow (VF) values of various measuring points.

	Measurement Point	COP	Sensitivity	Specificity	YI	PPV	NPV	Accuracy
PSV, cm/s	Inflow artery	≤96	41.7	81.6	0.233	41.7	81.6	72.0
		≤162	83.3	34.2	0.175	28.6	86.6	46.0
	Proximal 1/3 segment	≤90	54.5	80.4	0.349	57.1	78.7	72.0
		≤120	81.8	54.3	0.361	46.1	86.2	63.2
	Middle segment	≤55	40.9	84.0	0.249	52.9	76.4	70.8
		≤99	81.8	52.0	0.338	42.9	86.7	61.1
	Distal 1/3 segment	≤58	45.5	80.9	0.264	52.7	76.0	69.6
		≤104	81.1	48.9	0.307	42.8	85.2	59.4
	Outflow vein	≤49	23.8	83.0	0.068	38.5	70.9	64.7
		≤163	81.0	27.7	0.087	33.4	76.5	44.2
VF, mL/min	Inflow artery	≤206	75.0	83.3	0.583	60.0	90.9	81.2
		≤239	87.5	79.2	0.667	58.4	95.0	81.3
	Proximal 1/3 segment	≤175	38.9	81.0	0.199	46.7	75.6	68.4
		≤452	83.3	38.1	0.214	36.6	84.2	51.7
	Middle segment	≤195	42.1	80.4	0.225	47.0	77.1	69.2
		≤364	84.2	55.2	0.364	42.1	88.9	61.6
	Distal 1/3 segment	≤166	42.1	80.9	0.230	47.1	77.6	69.7
		≤339	84.2	42.6	0.268	37.2	87.0	54.6
	Outflow vein	≤105	42.1	81.4	0.235	50.0	76.1	69.4
		≤367	84.2	27.9	0.121	34.0	80.0	45.2

COP, cutoff point; NPV, negative predictive value; PPV, positive predictive value; YI, Youden Index.
